# Automated prediction of COVID-19 severity upon admission by chest X-ray images and clinical metadata aiming at accuracy and explainability

**DOI:** 10.1038/s41598-023-30505-2

**Published:** 2023-03-14

**Authors:** Alex Olar, András Biricz, Zsolt Bedőházi, Bendegúz Sulyok, Péter Pollner, István Csabai

**Affiliations:** 1 Department of Physics of Complex Systems, ELTE Eötvös Loránd University, Budapest, 1117 Hungary; 2 ELTE Eötvös Loránd University, Doctoral School of Informatics, Budapest, 1117 Hungary; 3 Data-Driven Health Division of National Laboratory for Health Security, Health Services Management Training Centre, Semmelweis University, Budapest, 1125 Hungary; 4 Department of Biological Physics, ELTE Eötvös Loránd University, Budapest, 1117 Hungary

**Keywords:** Medical imaging, Computational science, Machine learning, Statistical physics

## Abstract

In the past few years COVID-19 posed a huge threat to healthcare systems around the world. One of the first waves of the pandemic hit Northern Italy severely resulting in high casualties and in the near breakdown of primary care. Due to these facts, the *Covid CXR Hackathon—Artificial Intelligence for Covid-19 prognosis: aiming at accuracy and explainability* challenge had been launched at the beginning of February 2022, releasing a new imaging dataset with additional clinical metadata for each accompanying chest X-ray (CXR). In this article we summarize our techniques at correctly diagnosing chest X-ray images collected upon admission for severity of COVID-19 outcome. In addition to X-ray imagery, clinical metadata was provided and the challenge also aimed at creating an explainable model. We created a best-performing, as well as, an explainable model that makes an effort to map clinical metadata to image features whilst predicting the prognosis. We also did many ablation studies in order to identify crucial parts of the models and the predictive power of each feature in the datasets. We conclude that CXRs at admission do not help the predicting power of the metadata significantly by itself and contain mostly information that is also mutually present in the blood samples and other clinical factors collected at admission.

## Introduction

The main issue with the COVID-19 pandemic—or any pandemic for that matter—is that healthcare systems are not designed for abrupt increase in the influx of severe cases. When this happens the hospitals fill up quickly without being able to provide the necessary care for every individual. Due to the high pressure on healthcare workers semi-autonomous systems are always welcome in the assistance of decision making. The *Covid CXR Hackathon—Artificial Intelligence for COVID-19 prognosis* https://ai4covid-hackathon.ing.unimore.it/ challenge was launched and run for almost an entire month with this in mind. The goal of the competition was two-fold: whether it is possible to predict COVID-19 severity (*mild* or *severe*) by CXR (chest X-ray) images produced at admission with the accompanying clinical data from blood samples and other pre-screeing factors effectively; and whether such a high performance model could be made explainable. The challenge aimed at balanced accuracy, however we evaluated our models against other metrics as well. We explored several ideas and systematically built our models to include clinical data and extra features obtained by following the insights of other COVID-19 lung studies. Compared to previous similar works where the goal was to correctly classify COVID-19 cases against different types of pneumonia^1^, our task was considering whether it would be possible to predict the severity of COVID-19 based on initial CXR screening alongside the clinical metadata. Obviously this is also affected by many other factors, so we didn’t expect overly confident predictions as it was previously showed by others, what is approximately achievable on such data^2–4^.

Studies have explored the use of radiography for detecting and assessing the severity of COVID-19. Imaging modalities, such as chest X-ray (CXR) and chest computed tomography (CT), have been compared for their effectiveness in diagnosing and assessing COVID-19^5–7^. CXR was found to have lower sensitivity but has advantages over CT, such as lower cost, less need for patient transport, and lower radiation dosage. One study^8^ used synthetic X-ray images to validate their model, while two reviews^9,10^ analyzed CXR’s relationship with COVID-19 pneumonia.

In our work we have relied heavily on the Brixia-scoring system^2^. The Brixia semi-quantitative scoring system was first introduced at the beginning of the pandemic in order to aid radiologists with visual assessment and numerical scaling of the presence and severity of lung involvement amid the wide range of possible manifestation of pulmonary diseases. The prognostic value of the Brixia-score is further reinforced by other works^7,11^ showing that both the Brixia-score registered at admission and the highest Brixia-score registered during the hospitalization of a patient correlates with patient outcome.

Studies suggest^8,12^ using artificial data for COVID-19 imaging datasets, which are small compared to current deep learning datasets. However, this was not feasible in our case due to the presence of metadata and lack of a combined model to generate both COVID-19 CXRs and clinical features. In this regard, our task is also related to multi-modal learning with (CXR, free text diagnosis) data pairs, but we aim to learn from different modalities, not correspondence between diagnosis and CXR representations. Some work has proposed patch-based evaluation^13^ of CXR images, similar to the Brixia-scoring system. Severity prediction on CXR images without metadata has been done for COVID-19 and pneumonia^14,15^, but the severity metric was determined by radiologists, not disease outcome. There is also work discussing the differentiation of COVID-CXRs from regular CXRs^16^ and concluding for the need of more data. Studies have compared and evaluated popular CNNs^17,18^ for COVID-19 CXRs through accuracy metrics and input manipulation, some incorporating multiple datasets^4,19^ and highlighting common pitfalls^20^. Pre-training CNNs with novel datasets^21^ would have been also possible, however, we haven’t explored this, but used ImageNet pre-trained weight at initialization.

## Data

The data was provided by the challenge organizers and is available upon-request https://ai4covid-hackathon.ing.unimore.it/data (archived version: https://web.archive.org/web/20220924165541/https://ai4covid-hackathon.it/data), it includes a curated, raw dataset gathered from six Italian hospitals during the first SARS-CoV-2 outbreak in Italy (March–June 2020). All patients were confirmed to have contracted the disease and later on classified with a prognosis of severe or mild outcomes, this is considered as the ground truth (GT) in our study. A severe case meant that the patient needed mechanical ventilation or died, all other cases were considered mild. In addition to the imagery data, clinical metadata were provided for all the patients. Since not all the clinical features were available for each patient, the clinical data required a lot of data imputation before it could be used for modeling. Most of the data had been available before^3^ but the challenge organizers expanded it with several new data points. We have examined 1103 patients in this study, which included an additional $\sim$ 33% of data compared to the previous study^3^. The held-out test set was an additional 486 samples that we didn’t use for training but pre-processed the same way as the training data.

### Pre-processing

The images were converted from different types of DICOM images ranging from 12-bit to 16-bit in precision and also digitized in various ways. The resulting images which built up the training dataset varied significantly in terms of quality: some images were rotated randomly by 90$^{\circ }$, many were inverted and some contained a fully blacked-out or a gray margin on some edges of the scan. In order to deal with this, we selected the top two corners and the middle section of the scan in order to automatically detect whether the scan is inverted or not based on the mean brightness ratios between these regions; the inverted images have been reverted. We also manually annotated all images for inversion and concluded that our method was around 99% accurate. The left out images after the inversion method were corrected manually both in the training and the test sets. After finishing the inversion we followed with the pre-processing steps^2^ by applying clipping between the 2nd and 98th percentile of pixel values, quantizing the images to 8-bit, using contrast limited adaptive histogram equalization (CLAHE) and median filtering to reduce noise. Finally we scaled the images to 512 x 512 pixels in order to be appropriate for the BSNet^2^ network that we used to align, segment and score the images with the Brixia-scoring system.

### Noise reduction and data imputation

**Figure 1:**
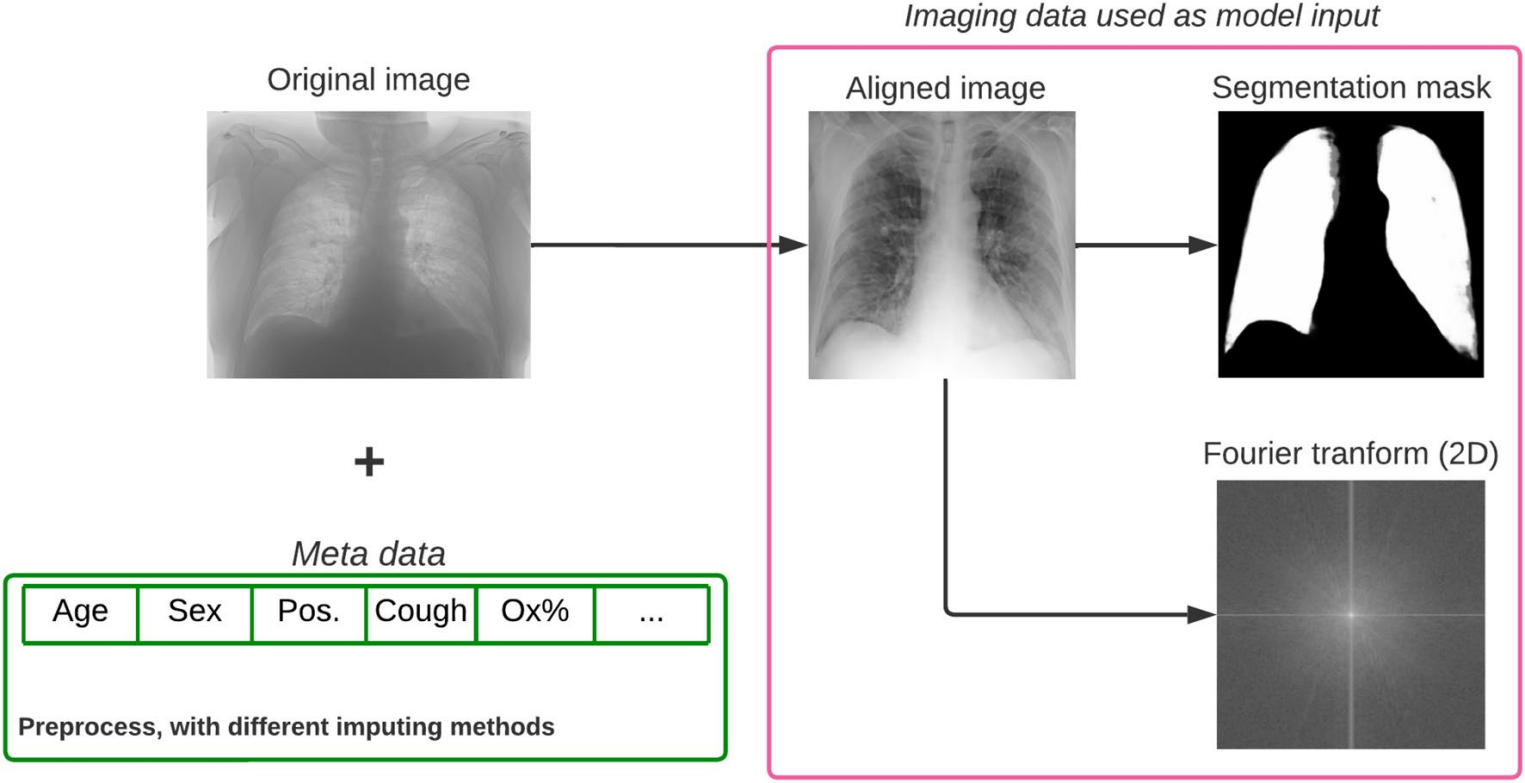
Original input images highly varied in brightness, contrast, pixel distribution, some were inverted and contained other artifacts. We manually checked and corrected them and also applied BSNet to align and segment them after the normalization process. The metadata was also standardized and different imputation methods were applied.

Having the pre-processed images, next we applied the BSNet^2^ in order to acquire their Brixia-scores, as well as aligning them and segmenting the lung area. The alignment played a crucial role in this project since the original images differed significantly, not only from hospital to hospital, but also within the same hospital, as a result, this process ensured that the images looked more uniform than they originally did (see Fig. 1). The segmentation on the aligned images was made more reliable by feeding in also the horizontally flipped counterpart of each image, then applying horizontal flipping again (flipping it back) on the output segmentation map and finally, averaging these two predicted outcomes. This way we averaged out the predictions, achieving better (lower variance) segmentation maps (not binary) for each aligned image and boosted the accuracies on hard-to-predict areas. We applied several pre-processing steps (whitening, one-hot encoding for hospitals, etc.) for the clinical metadata too and used different imputation methods to handle the missing values. These attempts contained imputation based on age groups, hospitals, population means (modes for categorical values) and random sampling the feature distributions of the present values. For further information regarding the feature types, counts and names please see Tables S1 and S2. The extended clinical dataset with the pre-processed images was used for the training both for the performance and for the explainable models. We also included the absolute value of the 2D Fourier-transforms of the aligned images in order to capture texture sensitive features with our image-only and performance models. All models were trained with five-fold cross-validation and hyperparameter optimized with Bayesian methods. The test set was held-out until the end of the challenge, therefore we handled it as a separate entity here as well.

## Methods

The authors confirm that all methods were carried out in accordance with relevant guidelines and regulations. The authors confirm that all experimental protocols were approved by the hackathon organizing team. Informed consent was obtained from all subjects or their legal guardians according to the local regulations of the hackathon organizers (*COVID CXR Hackathon—Artificial Intelligence for COVID-19 prognosis: aiming at accuracy and explainability*—https://ai4covid-hackathon.ing.unimore.it/prizes/).

We have systematically went through input modalities with different model classes in order to answer whether chest X-ray screenings at admission aid clinical metadata also collected at admission. We know from the literature^2,3,22^ that prognosis prediction from clinical indicators is possible, therefore we set our baseline to be the best classical machine learning model that can also be explained and reasoned about. We then took imaging data and attempted to also predict the prognosis without clinical metadata. Moving on we set up a precision network, building on top of our image only model to also process the clinical features and integrate that information into our prediction as well. We do this in a multitask learning setting^23^, also predicting death as a binary output for the image only and precision networks to aid predicting power. Finally, we built a separate, interpretable model that *reasons* about clinical features in the form of attention maps on the image.

To sum up, we can categorize our experiments into two sets: explainable and non-explainable. In the first category, we have our baseline models for the metadata only, we also have this, with the additional Brixia-scores for each sample. Also in this category is our precision neural network which incorporates clinical metadata and images. Moving on to the explainable category, we have the baseline models with the Brixia-scores evaluated for SHAP scores and the explainable neural network that generates visual interpretations alongside its predictions (see Fig. S2 in the “Supplementary material”).

### Classical baselines on clinical metadata

We evaluated several imputing methods, as our source training data was heavily corrupted by missing columns in specific hospitals, missing values for specific patients, etc. We turned to population sampling, population averages and also average imputing based on age groups as well as hospitals. We compare here these results between baseline models and as a result build an intuition on which imputing method could perform best in the long run for this specific dataset. The models were chosen based on popularity and general predictive power. We also explore whether adding additional information in the form of chest X-ray screenings at admission helps the predictive power or not.

### Precision network

Having acquired additional features in the form of 2D Fourier transforms, Brixia-scores, segmentation masks and aligned images as well as the imputed clinical metadata, we created a precision network, that integrates all these input features into one single unit with several single-feature processing layers/heads. A convolutional backbone processed the pre-processed image and the segmentation mask and after an average pooling created a feature vector out of the visual input feature. Meanwhile, another smaller convolutional network processed the Fourier features into a Fourier-feature vector, whilst a fully connected network processed the imputed clinical metadata concatenated with the Brixia-scores. All the output feature vectors of these single-feature processing units were then concatenated together and passed through a fully connected head that did the prognosis prediction in addition to the death prediction in order to do enhance the precision of our predictions. In the inference phase we only used the prognosis output to predict the severity of COVID-19 outcomes for the test set. The major parameters, such as the type of the convolutional backbone, size of the last dense layer used before the classification output, batch size, number of epochs, learning rate, etc. (see Supplementary Fig. S2 for the full list of parameters) were tunable hyper-parameters for which we did hyperparameter-optimization.

**Figure 2:**
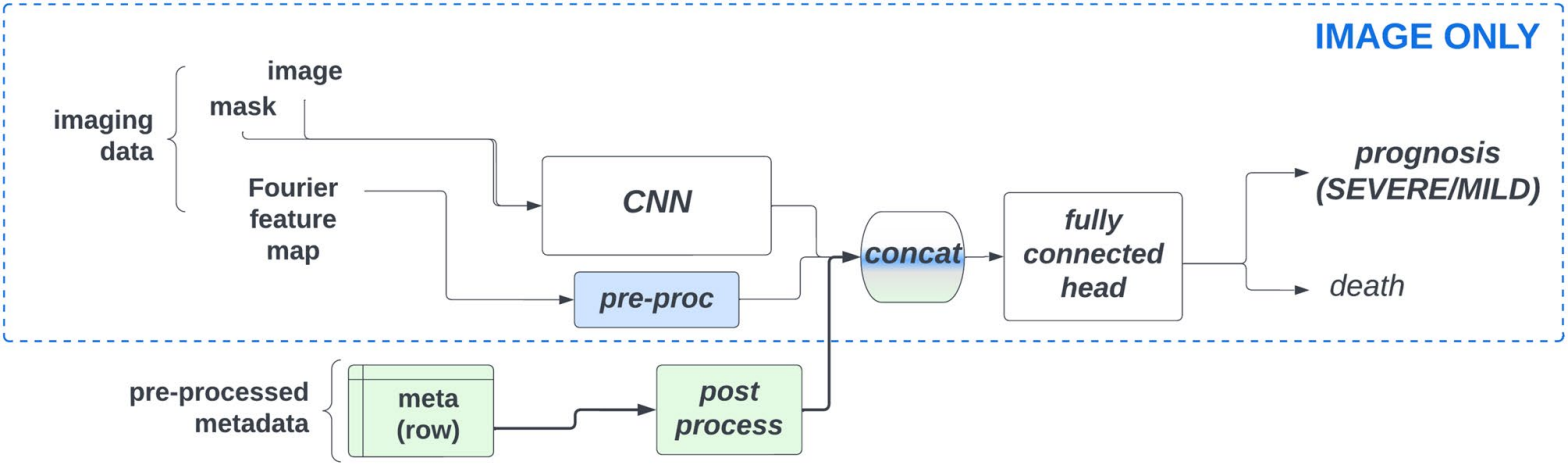
Our baseline model of the task at hand; the ’image-only’ model differs from this solely in the fact that the metadata input is not present, and not concatenated after post-processing to the other imagery based inputs.

We optimized the hyper-parameters for several models: (1) with image data only, (2) the image and metadata combined, (3) also we compared to the baseline models which were fitted on the metadata only (4) as well as the same baseline models with additional Brixia-scores. We have compared the performance of various architectures for each data modality.

### Explainable models

There are multiple ways to talk about explainability. We can try to *explain feature importances* of baseline models with statistical methods or think of the model as a black box, but create it as such, that it *outputs its own explanation*. First, we looked at two baseline models that performed well in our baseline comparison and added the Brixia-scores to the meta features to introduce information from the imaging data into the explainable models. This falls into the former explainable model category, reasoning about feature importances regarding the prediction. We have created the explaining plots with the SHAP library^24,25^ and selected the logistic regression and the XGBoost models for the explainable baselines as these are the most popular choices amongst machine learning experts for initial evaluation and are also easy to evaluate with the SHAP package. Afterwards, we experimented with an interpretable neural network, such that it generates visual interpretations of its prediction while still being a black-box predictor.

#### Visual interpretations

**Figure 3:**
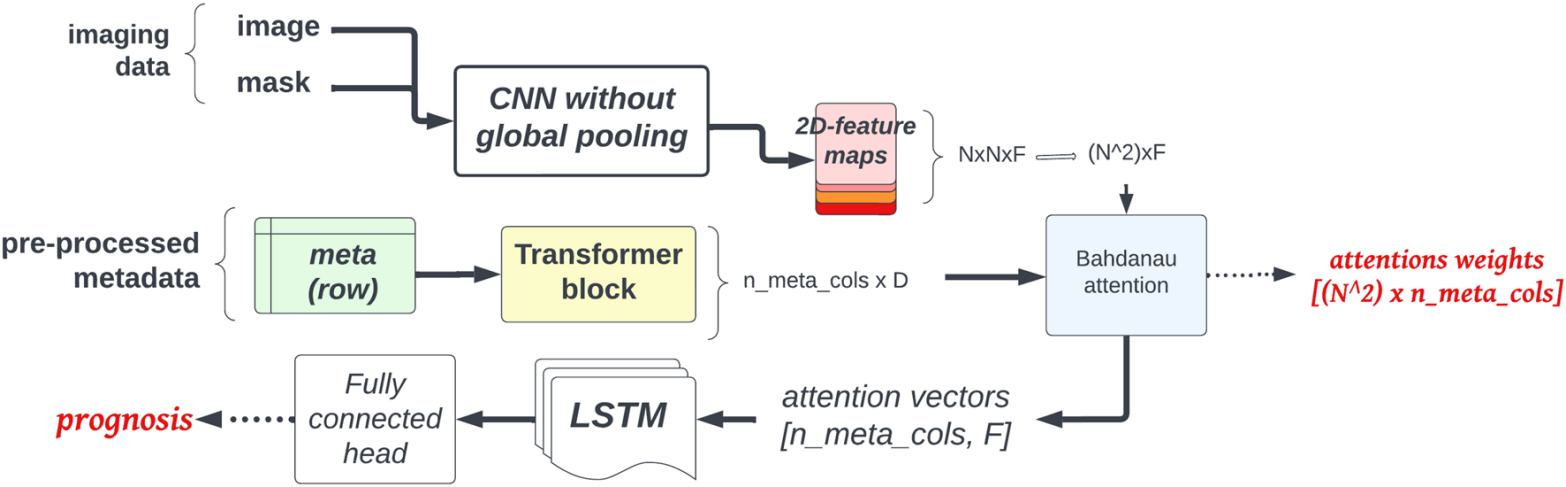
The explainable model generates explanations for its predictions via the output attention map created for each input image based on the metadata columns. Outputs are highlighted with red (prognosis and the attention maps over the input meta columns).

The explainable network has two main features, one convolutional sub-network processes the image data into an unpooled feature map ($16 \times 16$ or $8 \times 8$) with many feature channels. The second sub-network is a small transformer block^26^ for the clinical metadata. Afterwards, a Bahdanau attention map is calculated between each processed, embedded feature (meta vs. image) and are stacked together into one large context vector, averaged along the feature maps spatial dimensions after applying the attentional weights (see Equation 1). We process the context vector (still being a high-dimensional feature vector for each meta column) further with two layers of bidirectional LSTM networks^27^ having it only outputting prediction of the prognosis to model the *P*(*prognosis*|*image*, *meta*) distribution (see Fig. 3 for details). This model is optimized via stochastic gradient descent. During validation we tune hyper-parameters for the convolutional backbone, number of attention heads, dimensionality of the Bahdanau attention mechanism^28^, transformer encoding dimensions.

$$\begin{aligned} attention\_weights_{\ i} = SOFTMAX\Big (\mathbf {V_{i}} \times TANH\big ( \mathbf {W_{i}}^{image} \times encoded\_image + \mathbf {U_{i}}^{meta} \times encoded\_meta\_feature_{i} + \mathbf {b_{i}}\big )\Big ) \end{aligned}$$

**Equation 1.** Attention weights across the spatial dimensions of the encoded input image are calculated for each encoded meta feature. There are learnable weights $\mathbf {V_{i}}, \mathbf {W_{i}^{image}}, \mathbf {U_{i}^{meta}}$ indexed by ${\textbf{i}}$ which corresponds to a specific meta feature. This setup makes it possible to learn different attention weights for the same image but a different input meta feature.

Every neural network model was trained with the Adam optimizer^29^ and exponential learning rate decay. We optimized the hyper-parameters for the validation balanced accuracy metrics with $W \& B$^30^. Hyper-parameters and specific configuration files can be found on the project’s GitHub repository https://github.com/csabaibio/ai4covid/tree/feature/paper-changes.

## Results

**Figure 4:**
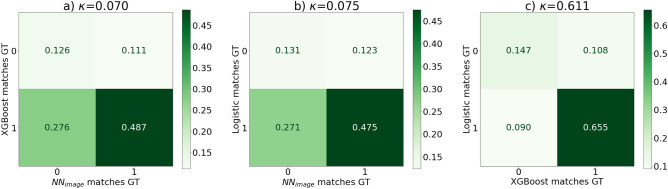
(**a**) XGBoost baseline model agreement with the ground truth against the image-only neural network ($NN_{image}$) agreement with the ground truth (GT), we can see that the XGBoost model is superior (**b**) shows similar result, as the logistic model agrees with the ground truth whilst the $NN_{image}$ model does not and the $NN_{image}$ model performs poorly also when the baseline models are wrong, (**c**) shows the baseline models against each other to see that similar prediction information content models miss and match together. All model comparisons have their Cohen-kappa scores calculated above the corresponding confusion matrices. We can see that the image-based networks have very low agreement with the baseline models. The numbers in each segment represent the fraction of cases corresponding to the specific match.

**Figure 5:**
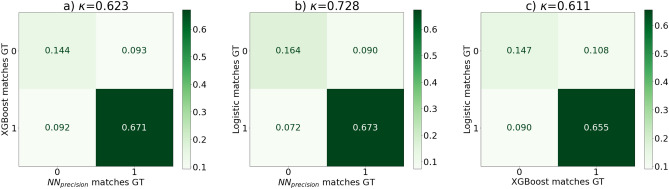
Compared to Fig. 4$NN_{precision}$ here stands for our *image + meta* model which shows great agreement with the baseline models, even more so, (in most cases) than the baselines agree with each other, this is also reflected by the Cohen-kappa^31^ scores.

Here we present our results featuring our baseline models, our precision network and later on we explore our explainable and our interpretable model in this scenario. We won *first place* [the first place was actually awarded to the authors of the baseline paper^3^ who resigned from the competition as they were data owners themselves] in the precision category of the challenge, and here we further explore our explainable model and look into additional explainable methods. We argue that the information contained in the CXRs at admission is present in the metadata, we tested this proposition via the trained models: we evaluated the agreement of our predictions of the logistic regression, XGBoost, image-only neural network and precision neural network models against the true labels. Creating this agreement output of each model (*1* if agrees, *0* if it does not), we could create confusion matrices of these agreement vectors between the models. If two models have similar output structure: one predicts the same examples severe/mild and misses similarly, their confusion matrix will be mostly diagonal. On the other hand, if a model is superior to the other, it is agreeing with the ground truth labels more whilst the other is being wrong. We can see the latter on the first two figures of Fig. 4, where we compare the logistic and XGBoost models against the image-only neural network’s outputs. Moving on to our precision network, we show that its predictions match the baseline models extremely well, which is presented in Fig. 5.

### Classical model performance on clinical metadata

We compared balanced accuracy, F1-score and AUC (area under the receiver-operator characteristics curve in Table 1 (and Supplementary Fig. S1). We found that the best performance was achieved with population average. We also validated this later on, with our precision network, and explainable network as well. The results were calculated with 5-fold cross-validation on the training data without hyperparameter tuning.

**Table 1:** Classical machine learning model performance on training data, on average the SVM model family performs the best during the cross-validation phase. Checking the held-out test performance of each model class, we can see in contrast to the cross-validation phase, in generalization (performance gap on unseen data) the logistic model is actually the best.

Model	Imputing	BA	F1-score	AUC score	BA (test)
Logistic	Population average	$0.745 \pm 0.019$	$0.756 \pm 0.023$	$0.834 \pm 0.012$	**0.763** ± **0.005**
	Population sample	$0.722 \pm 0.020$	$0.712 \pm 0.024$	$0.793 \pm 0.015$	$0.720 \pm 0.005$
	Age average	$0.732 \pm 0.019$	$0.733 \pm 0.024$	$0.810 \pm 0.017$	$0.757 \pm 0.005$
	Hospital average	$0.724 \pm 0.017$	$0.725 \pm 0.024$	$0.807 \pm 0.022$	$0.759 \pm 0.007$
SVM	**Population average**	**0.773** ± **0.014**	**0.787** ± **0.022**	**0.849** ± **0.020**	$0.737 \pm 0.005$
	Population sample	$0.697 \pm 0.026$	$0.700 \pm 0.023$	$0.791 \pm 0.016$	$0.716 \pm 0.009$
	Age average	$0.742 \pm 0.015$	$0.744 \pm 0.016$	$0.822 \pm 0.014$	$0.728 \pm 0.012$
	Hospital average	$0.729 \pm 0.029$	$0.737 \pm 0.035$	$0.809 \pm 0.026$	$0.734 \pm 0.007$
Random forest	Population average	$0.763 \pm 0.023$	$0.777 \pm 0.023$	$0.838 \pm 0.019$	$0.729 \pm 0.010$
	Population sample	$0.711 \pm 0.021$	$0.711 \pm 0.010$	$0.800 \pm 0.020$	$0.717 \pm 0.008$
	Age average	$0.720 \pm 0.013$	$0.721 \pm 0.020$	$0.807 \pm 0.010$	$0.728 \pm 0.012$
	Hospital average	$0.736 \pm 0.038$	$0.741 \pm 0.046$	$0.812 \pm 0.029$	$0.723 \pm 0.008$
Gradient boosting	Population average	$0.751 \pm 0.018$	$0.762 \pm 0.021$	$0.840 \pm 0.011$	$0.739 \pm 0.015$
	Population sample	$0.730 \pm 0.022$	$0.727 \pm 0.022$	$0.805 \pm 0.016$	$0.722 \pm 0.013$
	Age average	$0.750 \pm 0.019$	$0.750 \pm 0.022$	$0.832 \pm 0.017$	$0.724 \pm 0.005$
	Hospital average	$0.735 \pm 0.028$	$0.740 \pm 0.035$	$0.816 \pm 0.021$	$0.722 \pm 0.005$
XGBoost	Population average	$0.763 \pm 0.022$	$0.774 \pm 0.027$	$0.830 \pm 0.017$	$0.708 \pm 0.014$
	Population sample	$0.732 \pm 0.032$	$0.736 \pm 0.019$	$0.803 \pm 0.024$	$0.698 \pm 0.016$
	Age average	$0.734 \pm 0.019$	$0.735 \pm 0.022$	$0.827 \pm 0.016$	$0.701 \pm 0.010$
	Hospital average	$0.733 \pm 0.030$	$0.741 \pm 0.041$	$0.807 \pm 0.020$	$0.700 \pm 0.013$

Performance measure for the best model is noted in each column by using bold face numbers.

### Precision network results

We conclude from the results shown in Table 1 (and Supplementary Fig. S1) that the deep learning based models are on-par with the classical methods for this dataset and that the X-ray scans at admission do not improve the prediction of the prognosis significantly. Our best, cross-validated balanced accuracy score for the baseline models was ***0.773*** ± ***0.014*** with the SVM, meanwhile the best model on the held-out test set was the logistic model scoring a BA score of ***0.763*** ± ***0.005***, both highlighted in Table 1. Compared to the model solely with image based input data which included the lung mask score-map created with BSNet alongside with the 2D Fourier transforms of the input image (Fig. 2). We hyper-optimized the parameters of this network as well with the naive Bayes method (Supplementary Fig. S3) for the validation balanced accuracy score and concluded that our best overall BA score achievable via cross-validation was ***0.764*** ± ***0.020***, but as we know that the prediction problem has high variance, we could also do an ensemble prediction of the cross-validated models and evaluate the score with bootstrapping, in which case we achieved ***0.734*** ± ***0.021***. Both of these are below the scores of the baseline models, so it seems that the different input modalities actually hurt performance and there is no additional information in the early X-ray screenings. We sum up our findings in Table 2 and conclude that including the initial X-ray screenings into the predictive models is unnecessary and even detrimental for the accuracy of the prediction of the prognosis.

**Table 2:** Comparison of selected baseline models with the different input modality neural networks. All models were evaluated with population average based imputing. The selected explainable networks (logistic and XGBoost models with Brixia-scores included) are also shown in the table. The image based visual scores (Brixia-scores) do not significantly improve either generalization or model performance of the XGBoost or the logistic model. The precision network (combination of imaging and metadata) has much higher variance on the test set due to bootstrapping and it also shows that within our specific problem, slightly changing the test dataset, significantly impacts performance. In all the cases, except the neural networks, the test scores were acquired as the average outcome of the cross-validated models as opposed to bootstrapping (*) in the latter cases.

Model class	Balanced accuracy	F1-score	AUC score	BA (test)
Logistic regression	$0.745 \pm 0.019$	$0.756 \pm 0.023$	$0.834 \pm 0.012$	$0.763 \pm 0.005$
Logistic regression with Brixia-scores	$0.748 \pm 0.032$	$0.746 \pm 0.051$	$0.836 \pm 0.021$	$0.771 \pm 0.006$
XGBoost	$0.763 \pm 0.022$	$0.774 \pm 0.027$	$0.830 \pm 0.017$	$0.708 \pm 0.014$
XGBoost with Brixia-scores	$0.744 \pm 0.034$	$0.742 \pm 0.028$	$0.813 \pm 0.035$	$0.705 \pm 0.033$
Image-only neural network	$0.596 \pm 0.065$	$0.665 \pm 0.035$	$0.596 \pm 0.065$	$0.625 \pm 0.146$*
Precision neural network (image + meta)	$0.764 \pm 0.02$	$0.774 \pm 0.03$	$0.764 \pm 0.02$	$0.751 \pm 0.128$*

### Explaining the output

**Figure 6:**
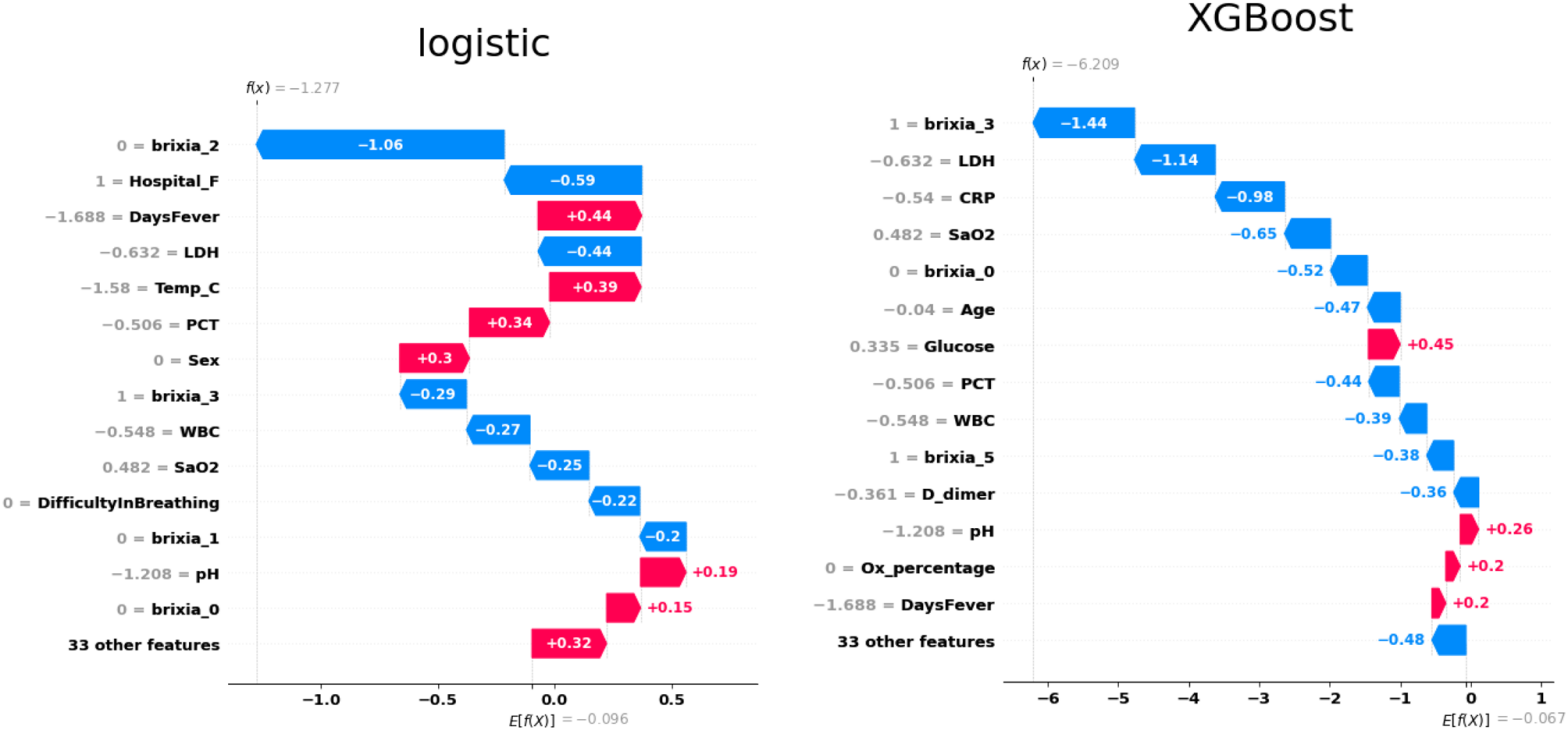
SHAP plots of the logistic (left) and XGBoost (right) models with the Brixia-scores; the expected model output is negative, therefore is biased towards the mild prognosis, we can see how relevant features contribute on average to either predicting a mild or a severe prognosis, from these plots we can see that the gender of a patient, the feverish days, pH levels, body temperature, age, being in a specific hospital, having a larger Brixia-score in a specific segment of the lung and some other factors influence the model towards a prediction of severe/mild prognosis.

Based on Fig. 6 (and Supplementary Fig. S4) we can conclude that the Brixia-scores, LDH (lactate dehydrogenase concentration in blood (U/L)) levels, being in a specific hospital, age, sex and body temperature of the patient are relevant features in generating the prediction of the prognosis. We can also see that body temperature, blood oxygen level and age also being highly influential, which we have expected (see Table 2). On the other hand, most of the remaining features are not highly affecting the prediction which is a good indicator of less predicting power.

For our explainable model we averaged the output attention maps of all images and metadata features both for the training and test samples to be able to spot any difference, and created mean attention maps for each relevant meta feature with respect to the predicted outcome. These attention maps correlate well with some of the metadata features, such as coughing, hearth failure, obesity, cardiovascular disease, etc.

**Figure 7:**
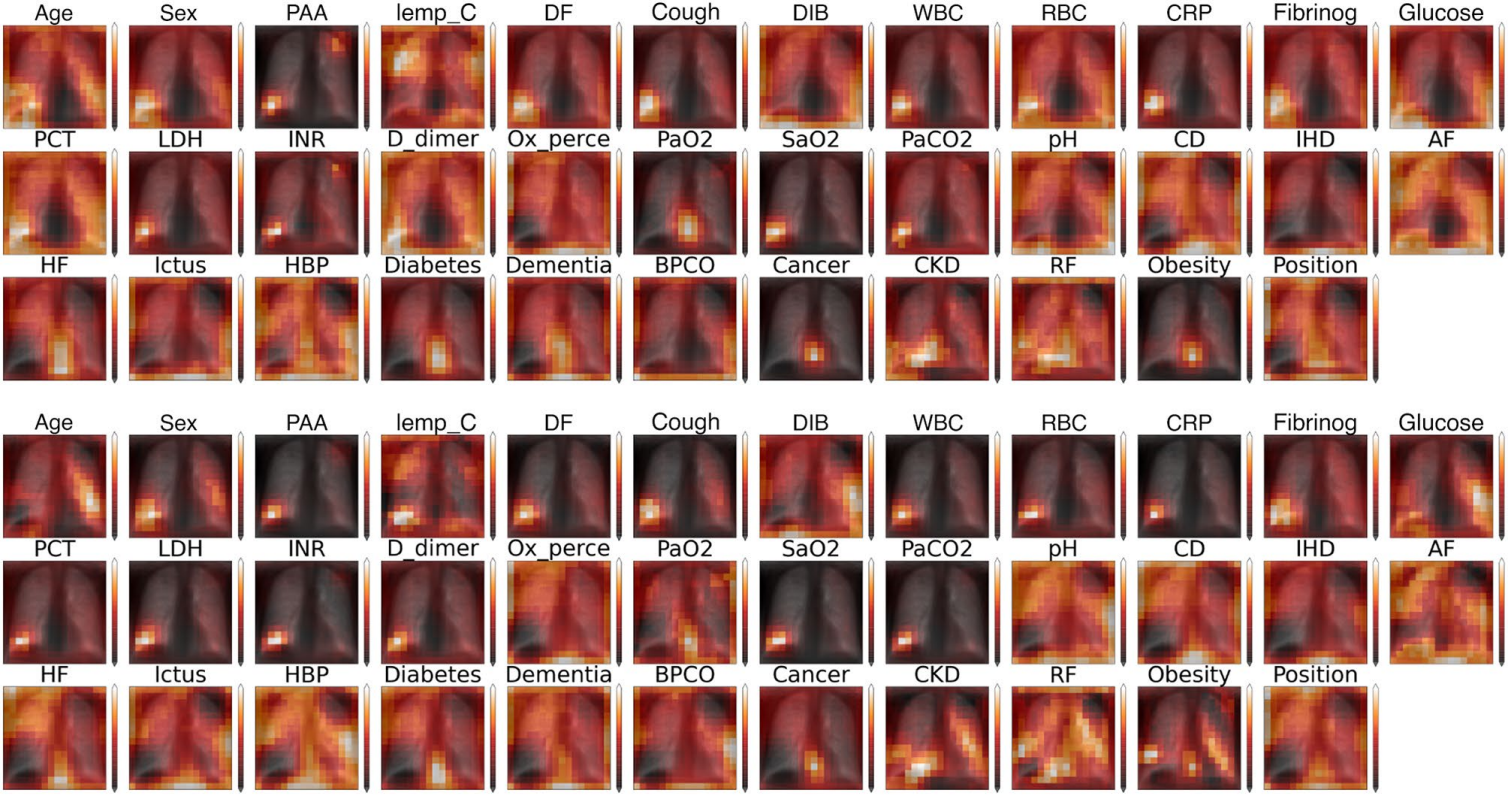
Individually scaled mean severe attention maps (top) and mean mild attention maps (bottom) on the test data. Here we can see high attention on the lower right lobe in both cases, with significant attention also on the heart and the region around it (HF—hearth failure, HBP—high blood pressure, CD—Patient had cardiovascular disease).

**Figure 8:**
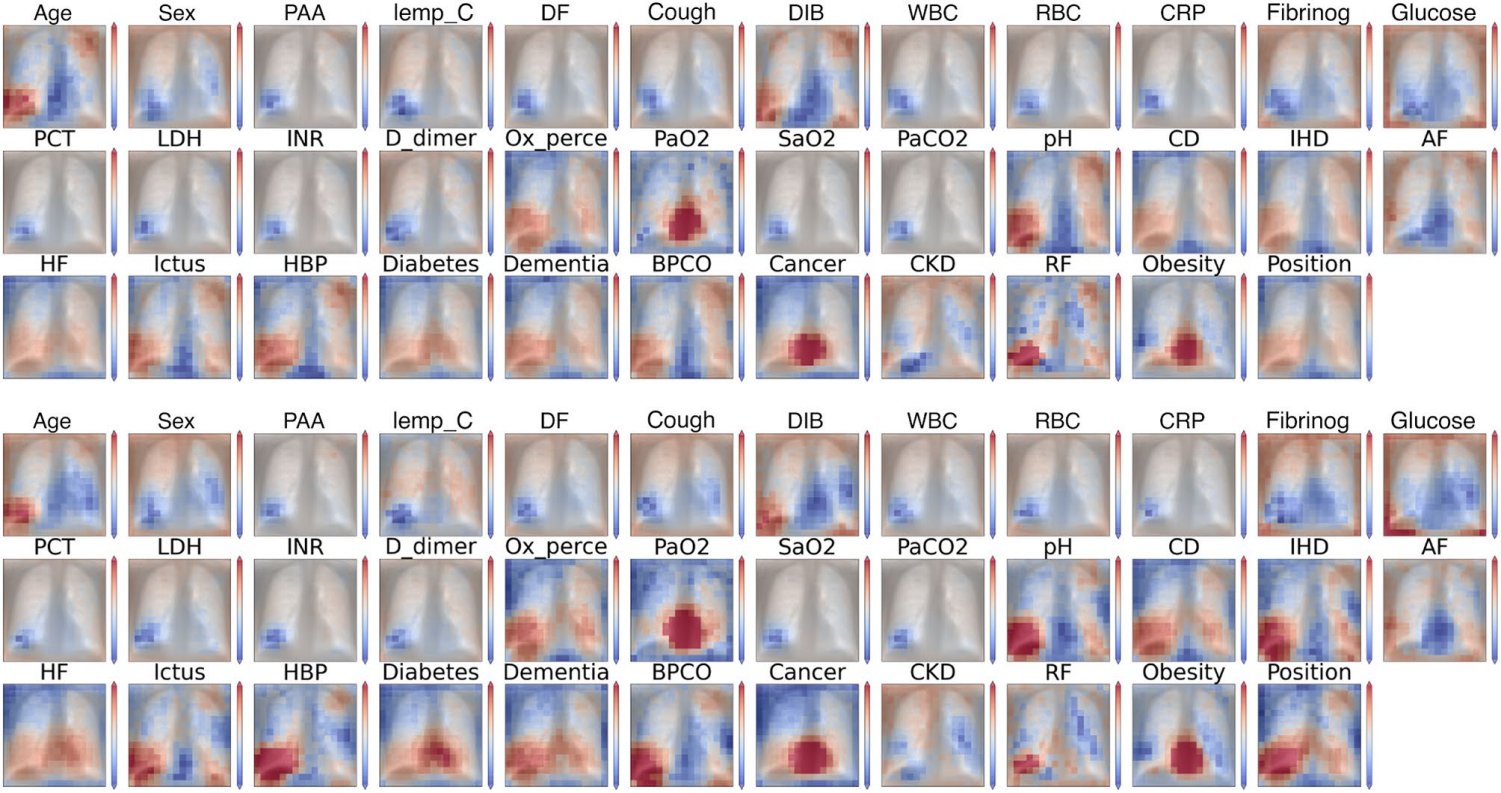
(top) Mean severe and mild prognosis difference attention maps on one of the validation sets (bottom) mean severe and mild prognosis difference attention maps on the test data, both having the mean lungs superimposed behind them. It can be seen that the average difference between severe and mild prognosis attentions is approximately the same for the validation and testing datasets, however, the test set distribution is not completely aligned with the validation set, therefore there are more extreme values in the attention differences. Being more red means higher severe attention, whilst being blue means a higher value of mild attentions. We can observe that given a severe prognosis, higher attention is put on the hearth and the lower lobes of the lungs in general with heart related metadata (HBP—high blood pressure, PaO${_2}$—oxygen levels, pH, obesity, IHD—ischemic heart disease etc).

We superimposed the mean severe and mild prognosis images behind the attention maps in Fig. 7. This way it can be seen in Fig. 8 that some features that are in connection with the hearth (e.g. hearth failure, cardiovascular disease) put high attention to that spatial region of the lung. Also the features that cannot be spatially localized in the attention maps tend to have a spread-out attention in the whole lung, such as obesity, dementia, glucose, days of fever etc. It also appears, that features which are highly correlated with the lungs themselves, have high attention on specific regions of the lungs (bottom or sides) as in the case of arterial blood oxygen pressure, oxygen percentage, difficulty of breathing, coughing, etc.

In Fig. 8 we show the same attention maps on one of the validation subsets and the test data to highlight that the model behaves the same way during validation and testing. We also found that taking the average Brixia-scores over severe and mild prognosis cases we get high correlation with the produced attention maps (Table 3). The Brixia-scores themselves function as a $3 \times 2$ matrix, scoring 6 segments of the lung, we up-sampled these to the size of our attention maps and calculated the absolute correlation between them with respect to the predicted prognosis by the network.

**Table 3:** The calculated correlation values were thresholded at a *0.2* absolute value in order to remove the less correlating features. The remaining features show above average linear absolute correlation with the Brixia-scores. Correlation variance was significantly reduced after the feature selection. The highly-correlating features are also shown in the table under the absolute correlation columns.

Data	Subset	Absolute correlation	Abs. corr. (thresholded)
Test	Mild	$0.200 \pm 0.105$	$0.288 \pm 0.062$
	Severe	$0.312 \pm 0.161$	$0.397 \pm 0.101$
Validation	Mild	$0.214 \pm 0.126$	$0.311 \pm 0.086$
	Severe	$0.265 \pm 0.151$	$0.369 \pm 0.075$
		*Obesity, high blood pressure, age, atrial fibrillation, ischemic heath disease, oxygen percentage in blood, D-dimer, dementia, respiratory failure*	

Among the linearly highly correlating features against the Brixia-scores (see Table 3) we can highlight the straight-forward ones which are: obesity, heart related meta features (high blood pressure, atrial fibrillation, ischemic heart disease, heart failure, etc.) which have high attention in similar regions where the Birxia-scores are high as well. We can conclude that these meta-features are even more important in the severe prognosis outcome, as they suggest initially worse conditions which eventually lead to a worse outcome of COVID-19.

## Discussion and conclusion

One very similar study to ours^22^ used a subset (cleaned data of advanced patients) of the same dataset and concluded that CXRs are actually a good indicator of mortality prediction. We argue that this is mostly explained by the selection criteria of patients in that study. The population in the study^22^ was very highly affected by COVID-19, and the patients were in a very late stage of the disease, so their initial CXR screenings already showed advanced signs of pneumonia and most of the lung area was affected. Another article^32^ found the CXRs to be an effective predictor of prognosis of COVID-19 in a population of patients aged between 21 and 50 years. We have not further explored this claim as their dataset contained much less patients (n = 145) and their study was based on different clinical parameters in a highly specific range. In this work, we have extensively studied whether CXRs at admission aid the prognosis prediction of patients for COVID-19 prognosis against clinical metadata collected upon admission by blood samples and other relevant factors that survey the patients. In contrast to the general assumption that gathering more modalities would always help, we found, that this does not hold true in the scenario when different modalities contain no additional information against each other. In this case, applying large models that operate with different modalities could be hard to train and can actually have detrimental effect on model performance due to the very hard task on information fusion. The problem of model degradation affects AI models in health care especially, because a few thousands or less patient data fall in the low data regime^33^. On one hand, our results do not validate previous results^3^ where the claim was to acquire marginal gains by data modality fusion. On the other hand, we also show, that such models are highly affected by the IID (independent and identically distributed) assumption, and varying the test set with bootstrapping, we get a much higher variance, as opposed to evaluating the same dataset, with a slightly different, cross-validated model (see Table 2). In conclusion, we state that initial CXRs do not possess additional information compared to the clinical metadata for the investigated population (see Fig. 5). This is due to, partly because of being in the low data regime and also because some relevant features are not yet developed at the time of admission in the lungs visually.

Our explainable model that generates visual interpretations shows intuitive attention maps, that might indicate medical insights about relations of the prognosis with visual cues. However, the lower right lobe area which got high attention could also indicate high variability in the input data around that region within males/females, age groups, etc. This also could hold true for the region of the hearth and the area around it, where there is *missing* information, since the hearth is covering up relevant regions of the lung. This reasoning is extremely hard to quantify, and is outside the scope of our study.

## Supplementary Information

Supplementary Information.

## Data Availability

The data that support the findings of this study are available from *Covid CXR Hackathon—Artificial Intelligence for Covid-19 prognosis: aiming at accuracy and explainability*—https://ai4covid-hackathon.ing.unimore.it/data but restrictions apply to the availability of these data, which were used under license for the current study, and so are not publicly available. Data are however available from the authors upon reasonable request and with permission of the challenge organizers. The pre-processing code is available in the code repository https://github.com/csabaibio/ai4covid/tree/feature/paper-changes. All experiments could be reproduced with the provided code samples and additionally generated information that is present there.
