# Supplementary material for: Automated prediction of COVID-19 severity upon admission by chest X-ray images and clinical metadata aiming at accuracy and explainability
Source: Sci Rep. 2023 Mar 14;13:4226. doi: 10.1038/s41598-023-30505-2 (PMC10012307; doi:10.1038/s41598-023-30505-2)
Supplement: Supplementary file 1 — Supplementary Information. [file 41598_2023_30505_MOESM1_ESM.pdf]

Supplementary material

The figures and tables below are supplementary to the article: Automated prediction of COVID-19 severity upon admission by chest X-ray images and clinical metadata aiming at accuracy and explainability.

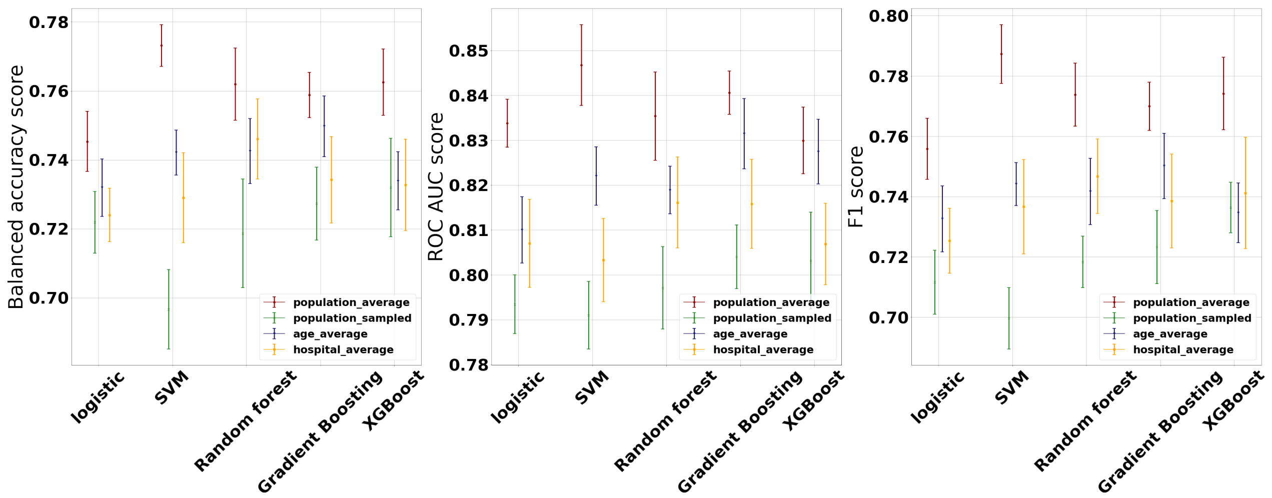

Figure S1. Comparison of baseline models across imputing methods and accuracy metrics; we can clearly see that during cross-validation population averaged based imputation performs the best across all model classes. We aimed at balanced accuracy (first sub-figure) but also validated this on AUC scores (second sub-figure) and F1-scores (third sub-figure).

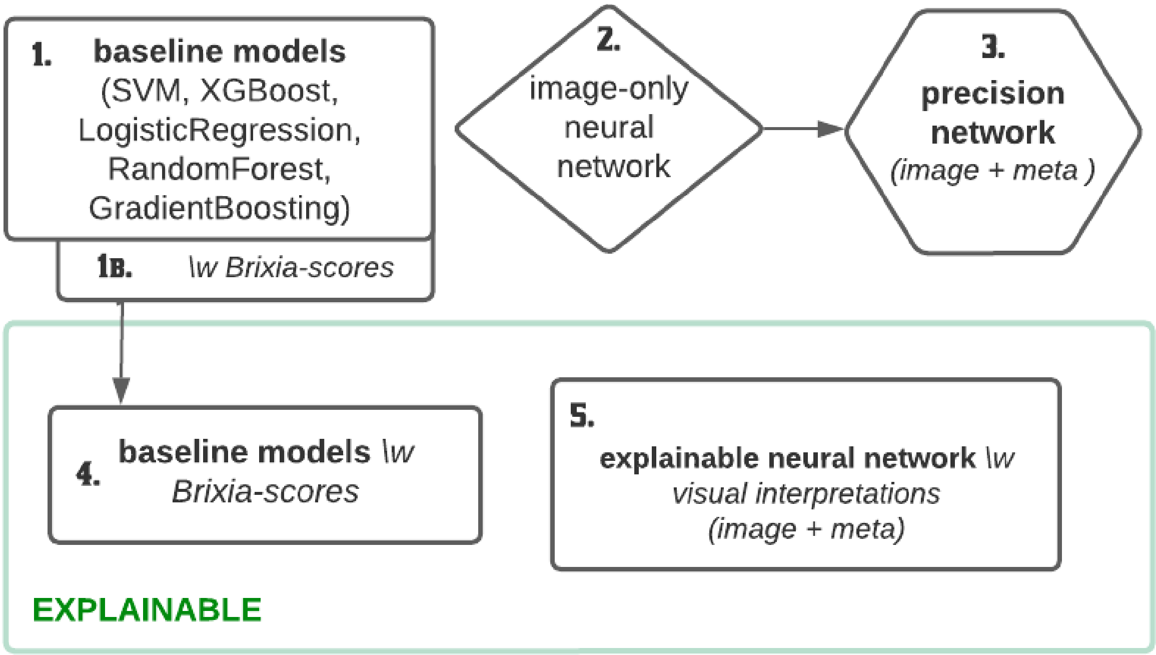

Figure S2. Diagram of the methods presented in this work.

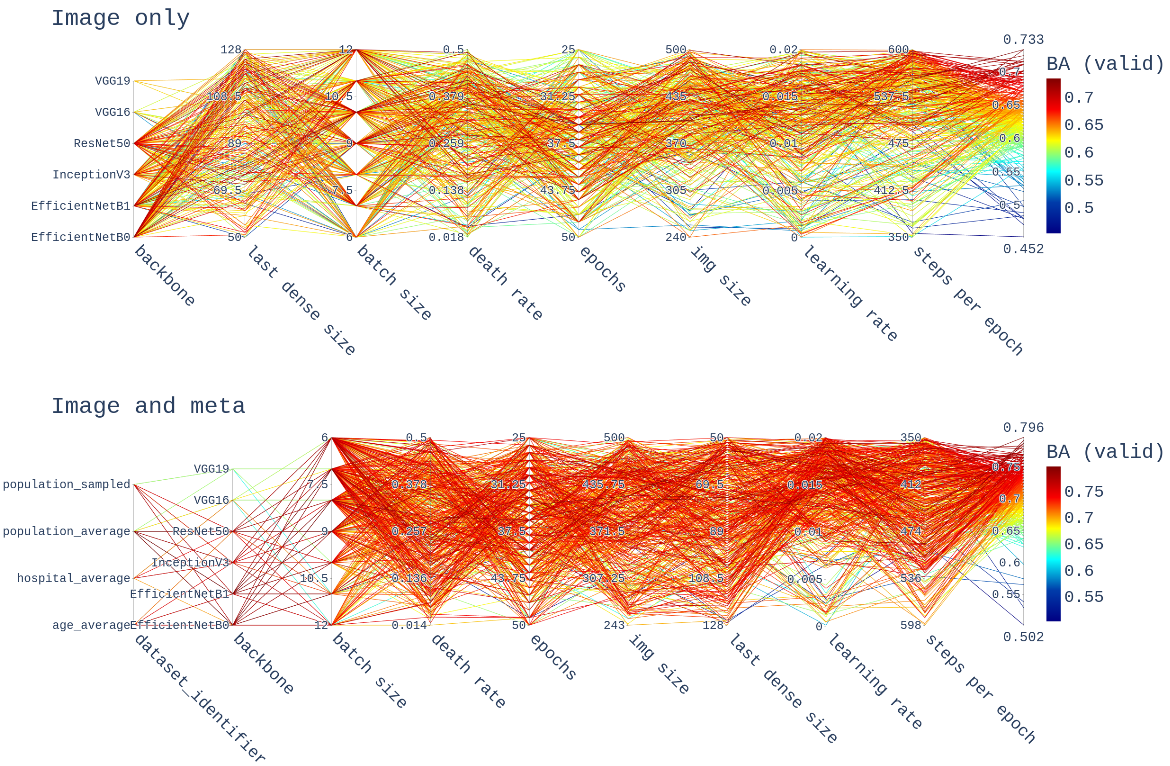

Figure S3. Hyper-parameter optimization of imagery based input network (top) and imaging data with meta-data based network (bottom), we can see from the optimization statistics that in the first case, the main parameter is the backbone network used, as opposed to the latter, the backbone parameter remains still important, yet the imputation method seems more crucial. BA (valid) - balanced accuracy on the validation set

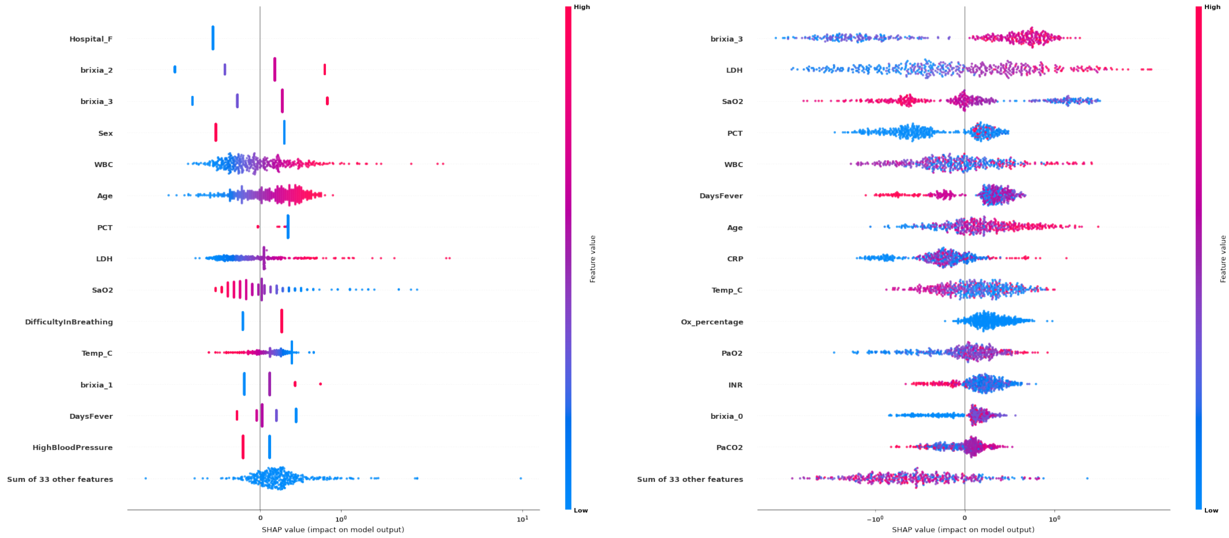

Figure S4. SHAP plots of the logistic model (left) and the XGBoost model (right) with the Brixia-scores, we can see the most relevant features explaining the output by sample values in descending order, we can see that the specific hospital, initial breathing difficulty, blood oxygen levels are very important in the prediction as well as some Brixia-scores, we can also observe that there is no crucial difference in the relevant feature subset (some Brixia-scores, age, sex, blood oxygen, etc.) Here a high feature value is associated with (has high impact on) the model output if it has a higher SHAP value, in a way we are looking for a form of correlation in the features and their importance for the prediction of prognosis for each individual sample.

Table S1. Categorical data, with total count (without NaN values), number of unique elements and the name-frequency pair of the top occurring value. The total dataset size was 1589 samples. The hospital where the data came from and the patients gender was always present, but it can bee seen that the counts of the other features are varying in numbers. To see a more detailed description of the dataset please check `https://ai4covid-hackathon.ing.unimore.it/data/`

	Hospital	Sex	PositivityAtAdmission	Cough	DifficultyInBreathing	CardiovascularDisease	IschemicHeartDisease
**count**	1589	1589	1550	1582	1583	964	829
**unique**	6	2	2	2	2	2	2
**top**	F	0	1	0	1	0	0
**freq**	974	999	1505	874	825	675	714

	AtrialFibrillation	HeartFailure	Ictus	HighBloodPressure	Diabetes	Dementia	BPCO
**count**	961	960	960	1446	1446	1443	1446
**unique**	2	2	2	2	2	2	2
**top**	0	0	0	0	0	0	0
**freq**	882	940	926	758	1225	1376	1350

	Cancer	ChronicKidneyDisease	RespiratoryFailure	Obesity	Position	Prognosis	Death
**count**	1446	1446	1304	1024	983	1589	1589
**unique**	2	2	2	2	2	2	2
**top**	0	0	0	0	1	MILD	0
**freq**	1346	1361	1281	934	791	841	1339

Table S2. Numeric data, the total dataset size was 1589 samples. While the age is almost always present for these patients we can observe that the other columns have a relevant portion of their features missing, hence we tried several imputation methods to check which work the best. To see a more detailed description of the dataset please check https://ai4covid-hackathon.ing.unimore.it/data/

	PaO2	RBC	WBC	Fibrinogen	Temp_C	SaO2	INR	pH
**count**	1155.00	1537.00	1563.00	279.00	1415.00	852.00	1279.00	1006.00
**mean**	72.82	4.58	6.97	600.43	37.33	92.90	1.27	7.45
**std**	25.19	0.69	3.63	157.50	0.99	6.37	2.95	0.11
**min**	17.60	0.26	0.20	102.00	35.00	29.00	0.04	4.53
**25%**	59.95	4.21	4.60	507.50	36.50	91.00	1.00	7.43
**50%**	68.70	4.60	6.21	602.00	37.30	95.00	1.08	7.46
**75%**	80.00	5.01	8.39	700.00	38.00	97.00	1.19	7.49
**max**	285.00	8.34	52.31	1171.00	41.50	100.00	105.00	8.02

	PCT	Glucose	Ox_percentage	Age	D_dimer	LDH	CRP	DaysFever	PaCO2
**count**	913.00	1382.00	739.00	1588.00	762.00	1251.00	1529.00	1179.00	1157.00
**mean**	27.55	125.84	92.63	65.64	2008.40	349.33	25.70	2.61	33.35
**std**	81.97	56.73	6.74	15.22	5200.57	205.78	53.94	1.06	5.95
**min**	0.02	22.00	50.00	17.00	5.61	0.02	0.01	0.00	16.90
**25%**	0.04	97.00	90.00	55.00	391.00	233.00	3.01	2.00	30.00
**50%**	0.12	111.00	94.90	67.00	707.00	307.00	9.00	3.00	33.00
**75%**	0.56	133.00	97.00	78.00	1455.00	404.50	19.70	4.00	36.20
**max**	571.00	874.00	100.00	100.00	54125.00	2903.00	570.50	4.00	92.30
